# The Frail-LESS (LEss Sitting and Sarcopenia in Frail older adults) intervention to improve sarcopenia and maintain independent living via reductions in prolonged sitting: a randomised controlled feasibility trial protocol

**DOI:** 10.1186/s40814-022-01225-7

**Published:** 2023-01-07

**Authors:** Daniel P. Bailey, Cherry Kilbride, Jamie H. Harper, Christina Victor, Marsha L. Brierley, David J. Hewson, Angel M. Chater

**Affiliations:** 1 Centre for Physical Activity in Health and Disease, Brunel University London, Kingston Lane, Uxbridge, UB8 3PH UK; 2 Division of Sport, Health and Exercise Sciences, Department of Life Sciences, Brunel University London, Uxbridge, UB8 3PH UK; 3 Division of Physiotherapy and Physician Associates, Department of Health Sciences, Brunel University London, Uxbridge, UB8 3PH UK; 4 Division of Global Public Health, Brunel University London, Uxbridge, UB8 3PH UK; 5 Institute for Health Research, University of Bedfordshire, Luton, LU1 3JU UK; 6 Institute for Sport and Physical Activity Research, University of Bedfordshire, Polhill Avenue, Bedford, MK41 9EA UK; 7 Centre for Behaviour Change, University College London, London, WC1E 7HB UK

**Keywords:** Sarcopenia, Frailty, Sedentary behaviour, Prolonged sitting, Behaviour change, activPAL

## Abstract

**Background:**

Sarcopenia is a progressive and generalised loss of muscle mass and function with advancing age and is a major contributor to frailty. These conditions lead to functional disability, loss of independence, and lower quality of life. Sedentary behaviour is adversely associated with sarcopenia and frailty. Reducing and breaking up sitting should thus be explored as an intervention target for their management. The primary aim of this study, therefore, is to examine the feasibility, safety, and acceptability of conducting a randomised controlled trial (RCT) that evaluates a remotely delivered intervention to improve sarcopenia and independent living via reducing and breaking up sitting in frail older adults.

**Methods:**

This mixed-methods randomised controlled feasibility trial will recruit 60 community-dwelling older adults aged ≥ 65 years with very mild or mild frailty. After baseline measures, participants will be randomised to receive the Frail-LESS (LEss Sitting and Sarcopenia in Frail older adults) intervention or serve as controls (usual care) for 6 months. Frail-LESS is a remotely delivered intervention comprising of tailored feedback on sitting, information on the health risks of excess sitting, supported goal setting and action planning, a wearable device that tracks inactive time and provides alerts to move, health coaching, and peer support. Feasibility will be assessed in terms of recruitment, retention and data completion rates. A process evaluation will assess intervention acceptability, safety, and fidelity of the trial. The following measures will be taken at baseline, 3 months, and 6 months: sitting, standing, and stepping using a thigh-worn activPAL4 device, sarcopenia (via hand grip strength, muscle mass, and physical function), mood, wellbeing, and quality of life.

**Discussion:**

This study will determine the feasibility, safety, and acceptability of evaluating a remote intervention to reduce and break up sitting to support improvements in sarcopenia and independent living in frail older adults. A future definitive RCT to determine intervention effectiveness will be informed by the study findings.

**Trial registration:**

ISRCTN, ISRCTN17158017; Registered 6 August 2021, https://www.isrctn.com/ISRCTN17158017

**Supplementary Information:**

The online version contains supplementary material available at 10.1186/s40814-022-01225-7.

## Introduction

Sarcopenia is a progressive and generalised loss of muscle mass and muscle function with advancing age [1]. In community-dwelling older men and women in the UK with a mean age of 67 years, the prevalence of sarcopenia was 4.6% and 7.9%, respectively [2]. A systematic review of adults aged ≥ 50 years, though, reported that the prevalence of sarcopenia in this population group could be as high as 29% [3]. This condition is associated with a range of health problems such as functional disability, falls, unplanned hospital admissions, poor quality of life, osteoporosis, cardiovascular disease, and premature mortality [4]. There is also a rapid rate of decline in activities of daily living (ADL) with 19–39% of community-dwelling older adults with sarcopenia becoming dependent on others for ADL over a 2-year period [5]. Sarcopenia is considered one of the main contributors to the development of frailty and is a key mediator in the pathway through which negative health outcomes of frailty arise [6]. Frailty can be defined as a clinical state in which an individual’s vulnerability for developing increased dependency and/or mortality when exposed to a stressor is increased [7]. This syndrome has multiple causes characterised by diminished strength, endurance, and physiological function that increases vulnerability to co-morbidities, dependency on others for ADLs, and death [7]. The risk of all-cause mortality, unplanned hospitalisation and nursing home admission over a 1-year period is increased by 92%, 93%, and 89%, respectively, in individuals with mild frailty [8]. Moderate and severe levels of frailty are associated with markedly higher risk of these outcomes [8]. It is thus important to develop interventions to reduce the progression of sarcopenia and frailty in older adults to support maintenance of independent living and reduce the risk of adverse health outcomes.

Reducing sedentary behaviour (i.e. any waking behaviour with an energy expenditure ≤ 1.5 METs while sitting, lying or reclined [9]) may be an appropriate initial intervention target for the prevention and management of sarcopenia and frailty. At this initial stage, sedentary time could be replaced and broken up with standing or light-intensity physical activity. This may set the foundations for progressing onto moderate-to-vigorous intensity physical activity, which is unlikely to be achievable to begin with for individuals with chronic disease or physical impairments [10]. A focus on reducing sedentary behaviour is supported by evidence that increased daily sitting time is negatively associated with muscle mass and physical function [11, 12] with each additional hour of daily sitting increasing the risk of sarcopenia by 33% in community-dwelling older adults [13]. Time spent engaging in sedentary behaviour was likewise associated with higher frailty and explained a greater proportion of variance in this outcome than time spent in moderate-to-vigorous physical activity [14].

In addition to total sedentary time, the manner in which sedentary time is accumulated also appears to be important for optimising health in older adults. A higher number of breaks in sitting (i.e. moving from sitting to standing or walking) is related to improved physical function, reduced difficulty in ADL, reduced frailty and a 45% reduced risk of sarcopenia [12, 15–17]. These health risks were independent of moderate-to-vigorous-intensity physical activity levels [15, 16], meaning that reducing and breaking up sitting with standing or light-intensity physical activity could be important intervention targets. Indeed, reducing and breaking up sitting is recommended in national and international physical activity guidelines for older adults and is particularly encouraged for frail older adults for whom more strenuous activities are less feasible [18, 19]. Interventions are thus needed to support reducing and breaking up sitting to improve sarcopenia, slow the progression of frailty and to maintain independent living.

The general older adult population spend 9.4 h a day being sedentary according to a systematic review of 350,000 participants, making them the most sedentary age group [20]. As older adults have the highest prevalence of chronic disease and consider interventions targeting sedentary behaviour to be more acceptable than structured exercise approaches due to barriers such as pain, fatigue, and risk of injury [21, 22], investigating the feasibility and efficacy of interventions to reduce sedentary time in community-dwelling older adults is warranted. An 8-week intervention consisting of motivational interviewing, goal setting, self-monitoring diaries, and mailed feedback (on sitting, standing, stepping, and sit-to-stand transitions at 1 and 3 weeks) significantly reduced sitting by 27 min per day in older adults who were overweight or obese [23]. Sitting time in this intervention was primarily replaced by standing, which increased by 25 min per  day [23]. A systematic review of seven randomised controlled trials (RCTs) in community-dwelling older adults found a mean reduction of 45 min per day in total sedentary time for sedentary behaviour interventions compared with controls, albeit there was insufficient data to pool the effects on breaks in sedentary time [24]. Due to limited evidence and small sample sizes, there was also uncertainty regarding the effectiveness of these interventions for improving physical and mental health, including physical function [24].

It remains unknown whether the intervention findings in older adults are generalisable to individuals living with frailty who have difficulty with ADLs and engage in significantly higher volumes of sedentary time [14]. An intervention in community-dwelling older adults with frailty (*n* = 23) that comprised of face-to-face motivational interviewing, physical function test feedback, plus visual and real-time feedback on sitting using a wearable device, reported significant improvements in breaks in sedentary time, physical function (timed up and go and sit-to-stand tests), and quality of life after 14 weeks [25]. There was, however, no change in daily sitting, which could be expected given this study was not powered to detect a change in this outcome [25]. This pilot study was also limited as it did not include a usual care control group and had a high dropout rate of 45% [25]. However, a powered RCT (*n* = 43) in older adults with frailty did report a 30 ± 10 min/day reduction in sedentary time after a 16-week intervention consisting of home-based standing exercises, face-to-face health education, and telephone support [26]. Nevertheless, there was no change in the number of breaks in sedentary time, and standing time or physical activity were not reported so it is unclear what activities sedentary time was replaced with. Furthermore, intervention effects on health and wellbeing outcomes were not evaluated [26].

Overall, the literature regarding interventions focusing on reductions in sedentary time in community-dwelling older adults is scarce and characterised by studies that are short-term (typically < 3 months), low quality, predominantly conducted with healthy participants and have lacked evaluation of health outcomes relevant to older adults, including sarcopenia-related outcomes [24, 27]. Investigating the feasibility, safety, and acceptability of an intervention to improve sarcopenia and independent living in older adults with frailty via reducing and breaking up sitting is therefore warranted. Furthermore, an intervention that is delivered remotely and is suitable for individuals who may be experiencing physical or social isolation (representing 7–17% of the older population [28]), or physical distancing due to location or for infectious disease control, has not been evaluated. This is particularly relevant for individuals with frailty who may have difficulty in getting outside of the home independently or are particularly concerned about minimising contact with people outside of their household, leading to increased sedentariness [29, 30]. The primary aim of this study is to examine the feasibility, safety, and acceptability of conducting a definitive RCT to evaluate a remotely delivered intervention to improve sarcopenia and independent living through reducing and breaking up sitting in frail older adults.

The main objectives are to:

- Determine an appropriate participant recruitment strategy;
- Evaluate participant retention and data completion rates for the primary outcome in a future definitive RCT;
- Assess acceptability of randomisation to study groups;
- Evaluate intervention acceptability, fidelity, and adherence;
- Determine trial safety.

The secondary objectives are to explore the potential efficacy of the intervention for improving sarcopenia, physical function, device-assessed daily sitting, prolonged sitting, breaks from sitting, standing and stepping, mood, wellbeing and quality of life**.**

## Methods

### Study design

**Fig. 1:**
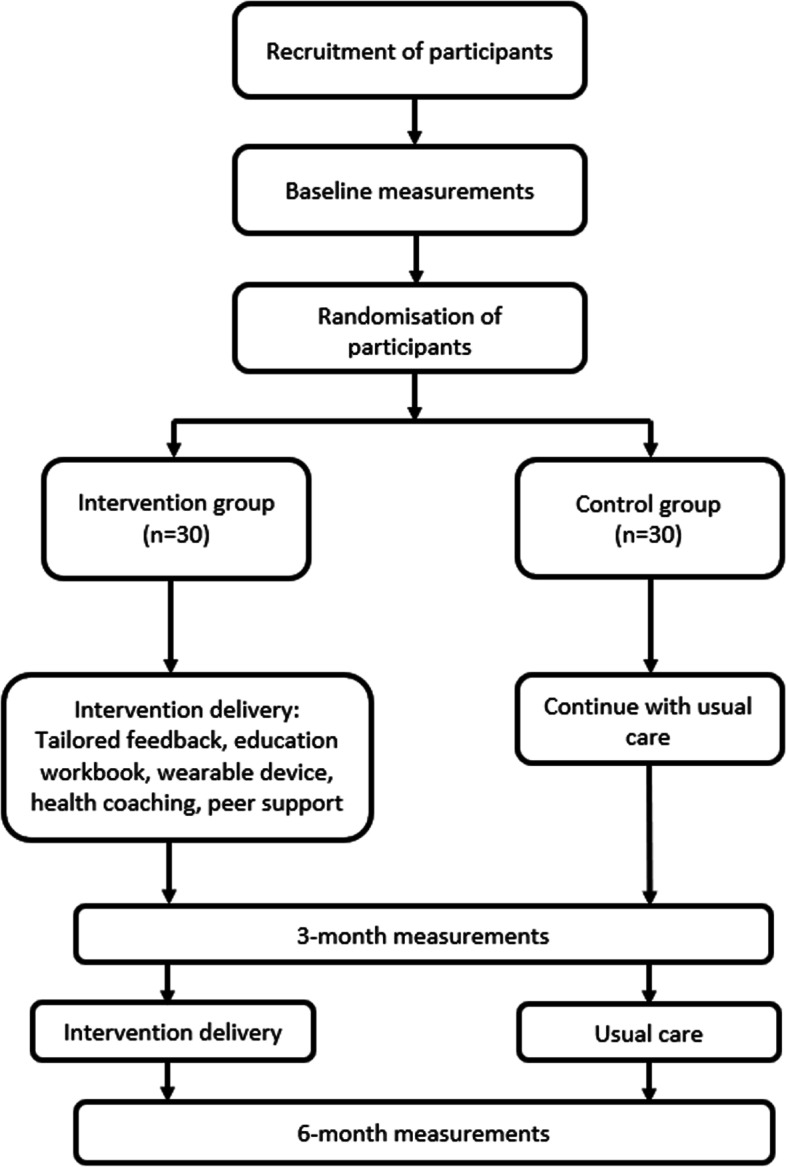
Study flow diagram

This is a randomised controlled feasibility trial that will be conducted and reported following the Consolidation Standards of Reporting Trials statement for pilot and feasibility trials [31]. The reporting of the study protocol follows the Standard Protocol Items: Recommendations for Interventional Trials statement [32]. After baseline measures, participants will be randomised to the intervention (Frail-LESS) or control (continue with usual healthcare) groups. Measurements will then be repeated 3 months and 6 months after group allocation. This intervention duration and the timing of measurements was deemed appropriate for determining the feasibility of the study and intervention acceptability. A flow diagram providing an overview of the study can be seen in Fig. 1.

### Study setting

This study will be conducted with community-dwelling older adults living in the London metropolitan area, England. Study measurements will be taken at Brunel University London or at participants’ homes depending on participant preference and ability to travel to the University campus. All components of the intervention will be delivered remotely.

### Participant recruitment

The recruitment strategy for the study will include GP practices sending out SMS text messages to potentially eligible patients in their databases. Database searches will be based on patient age, community-dwelling, and a classification of mild or moderate frailty according to an electronic frailty index (eFI) score of 0.13–0.24 and 0.25–0.36, respectively [8]. Database searches will include patients classified with moderate frailty by the eFI as they may be eligible for the present study according to the Clinical Frailty Scale eligibility criteria explained below [33]. The North West London Local Clinical Research Network will support recruitment from GP practices. Participants will also be recruited through Lindus Health, which is a registered UK company that provides recruitment services to health research and clinical trials. Lindus Health use paid advertising on social media (Facebook; Meta, CA, USA), which will be targeted at the North West London region. Email circulation to Brunel Older People’s Reference Group and a study webpage will also be used for recruitment. Potentially eligible participants will be asked to express their interest by email, phone, click-throughs on social media posts, or scanning a Quick Response code provided on recruitment materials. A researcher will then contact interested individuals by email and telephone to carry out screening. 

### Participant eligibility criteria

Screening for participant eligibility will be administered over the phone by a researcher with a clinical health professions background. Eligible participants for this study will be community-dwelling older adults aged ≥ 65 years living with very mild or mild frailty classified according to the Clinical Frailty Scale version 2.0 [33]. Very mild frailty describes people who are not dependent on others for daily help, but often have symptoms that limit activities; a common complaint is being “slowed up” and/or being tired during the day. Mild frailty describes people who have more evident slowing and need help in high order instrumental activities of daily living. Typically, mild frailty progressively impairs shopping and walking outside alone, meal preparation, and housework. Additional inclusion criteria are (1) participants spend the majority (> 60%) of the waking day sitting, measured via self-report using the International Physical Activity Questionnaire item (“During the last 7 days, how much time did you spend sitting on a week day?”) and (2) have a Functional Ambulation Category rating of ≥ 4, i.e. able to ambulate independently with or without a walking aid on level surfaces without supervision or assistance from another person [34].

Exclusion criteria include being unable to communicate in English to a level needed for effective engagement in the study, any physical or mental impairment affecting participants’ ability to stand and walk, and cognitive impairment (score ≥ 7 in the Six-Item Cognitive Impairment Test [35]).

Individuals who are identified as being eligible will be invited to provide written informed consent and to make an appointment to have baseline measures taken.

### Sample size

The target sample size is 40 participants (*n* = 20 in each the intervention and control groups), which is in line with sample size recommendations for pilot and feasibility studies [36, 37]. This number of participants is, therefore, sufficient for addressing the primary objectives of the study relating to feasibility, safety, and acceptability. To allow for 30% attrition, the study aims to recruit 60 participants.

### Randomisation

Randomisation will be undertaken using an online tool (www.randomizer.org) with a fixed block size of four to allocate participants on an individual basis at a ratio of 1:1 to the intervention (Frail-LESS) and control (continue with usual healthcare) groups. This approach will be used to increase the likelihood of balanced numbers in the trial arms. The randomisation will be conducted by a researcher independent from the study team. Researchers and participants will be blinded to group allocation until baseline measurements have been completed.

### The Frail-LESS intervention

#### Intervention development

The Frail-LESS (LEss Sitting and Sarcopenia in Frail older adults) intervention was developed following Medical Research Council guidelines for developing and evaluating complex interventions [38]. The Behaviour Change Wheel (BCW), which has the COM-B (Capability, Opportunity, Motivation-Behaviour) system at its hub, was used as a framework enabling translation of a COM-B diagnosis to the design of an evidence-informed intervention [39, 40]. Barriers and facilitators for reducing and breaking up sitting were identified from a systematic review and thematic synthesis of older adults’ perceptions of sedentary behaviour, which included 351 participants across 15 medium or high quality studies [22]. These were mapped to the BCW intervention functions and the policy categories through which those functions would be supported. Next, the optimal behaviour change techniques (BCTs) [41] for reducing and breaking up sitting and their mode of delivery were identified. This approach to intervention development has been used in previous cost-effective interventions that significantly reduced sitting and improved cardiovascular health and psychological wellbeing in office workers in the short and long-term [42–45] and within a community setting [46]. The proposed intervention was then refined following consultation among the research team and with the Brunel Older People’s Reference Group taking into consideration the APEASE (Acceptability, Practicability, Effectiveness/cost-effectiveness, Affordability, Safety/side-effects, Equity) criteria [47] and the ability for the intervention to be delivered remotely. The mode of delivery of the BCTs in this intervention will include the use of tailoring and technology (as described below), which older adults most frequently identified as being suitable to support reductions in sedentary behaviour [22]. A meta-analysis also reported significant reductions in sedentary time and improvements in physical functioning in response to technology-delivered behaviour change interventions in older adults [48]. However, some older adults indicated that technology-based interventions may not be acceptable due to issues such as lack of familiarity or access to electronic devices or the internet [22]. It is therefore possible that the intervention evaluated in this study may only be representative of older adults who volunteer to take part in the study, and who are familiar with technology and/or willing to develop their competence.

#### Intervention protocol

The Frail-LESS intervention will consist of tailored feedback, a psychoeducation workbook, a wearable device, health coaching and peer support, as described in detail below. Each participant randomised to the intervention group will receive the Frail-LESS intervention for 6 months. At the outset, participants will be posted an intervention pack consisting of a tailored feedback sheet, a psychoeducation workbook, a wearable device and accompanying user manual, an information sheet summarising the intervention (including contact details for their health coach), and information on how to join a peer support group. These materials (other than the workbook and wearable device) will also be emailed to each participant by a researcher. Specifically, the intervention components include the following:

##### Tailored feedback

Each intervention participant will be provided with a personalised feedback sheet based on their sitting, standing and stepping measurements at each time point (i.e. baseline, 3 months and 6 months). This will include a graphical representation and written explanation of their daily sitting, standing, stepping and number of breaks from sitting.

##### Psychoeducation workbook

This paper-based workbook will cover information on the health risks of sitting too much, potential benefits of reducing and breaking up sitting, tasks for goal setting, action planning, identifying potential barriers to reducing and breaking up sitting, and examples of activities to reduce and break up sitting.

##### Wearable device

A Garmin Vivofit wrist-worn device (Garmin Ltd., Kansas, USA) will be accompanied by a guidance document for its use and the manufacturer’s manual. The Vivofit tracks inactive time, daily steps and daily energy expenditure (kcal). A coloured move bar fills up on the display with an audible alert if the user has been inactive for too long to prompt them to get up and ambulate for a few minutes so the bar can be reset. The user can choose to connect the device to a smartphone app to set their own personal step goals and visualise a wider array of activity-related data.

##### Health coaching

Individual health coaching sessions will be provided throughout the intervention by individuals trained in coaching for health specifically for this study. The health coaches will have backgrounds in health psychology and behaviour change. Training developed and led by A.C. will cover how to identify influences on sitting behaviour in relation to capability, opportunity, and motivation to create a COM-B diagnosis [49, 50]. This will be facilitated with communication style motivational interviewing [51–53], and the GROW model of health coaching (GROW: goal, reality, options, will/way forward) [54]. Trained health coaches will follow the ‘engage, focus, evoke, plan’ strategy of motivational interviewing, with an emphasis on the consultation allowing partnership, acceptance, compassion, and evocation (PACE) [55]. Core communication skills to support an effective consultation [56, 57] will be used, such as the RULE (Resist the righting reflex; Understand client motivation; Listen; Empower) and the OARS (Open-ended questions, Affirmations, Reflective listening, Summaries), showing empathy, building rapport and rolling with resistance when needed. These will be linked to the delivery of relevant BCTs; this person-centred approach aims to increase self-determined capability, opportunity, motivation and ultimately behaviour. Health coaching sessions will take place 3 to 5 days after allocation to the intervention group followed by further sessions approximately 2, 6, 12, and 18 weeks after the intervention starts. Health coaching will take place via video call or telephone, based on participant preference.

##### Peer support

Intervention participants will be provided with the opportunity to join a Frail-LESS peer support group. A monthly virtual meeting will be arranged by the research team and will be structured in a way that encourages the participants who opt to take part to discuss their experiences of the intervention, suggest and ‘try-out’ different activities that could be used to reduce and break up sitting, and offer general support to one another. A WhatsApp group will be created by one of the intervention participants to provide participants with the opportunity to interact with one another outside of the virtual meetings. Participants will be advised they can also exchange email addresses to communicate with one another if they wish to.

### Control group

Individuals randomised to this group will continue with any usual healthcare that they may be receiving and not receive the Frail-LESS intervention. They will have the opportunity to receive tailored feedback based on their 6-month sitting, standing and stepping measurements, the psychoeducation workbook, wearable device and join the peer support group once the study has ended. Participants in this group will undergo the same set of measurements as the intervention group at all time points.

### Measurements

#### Trial feasibility outcomes

Feasibility outcomes will include participant eligibility, recruitment and retention rates for the trial. Data completion rates for the intended primary outcome in a future trial (sarcopenia) will also be assessed. Acceptability of the intervention to participants, intervention fidelity and adherence will be evaluated during the process evaluation as described below. Intervention safety will be evaluated in terms of pain (using a 100-mm visual analogue scale [58]) and fatigue (using the Fatigue Severity Scale [59]) in addition to frequency of falls, hospital admissions and adverse events at each data collection time point. Adverse events will also be recorded ad-hoc if reported by a participant.

#### Demographic information

At baseline, demographic data will be collected including age, sex, ethnicity, comorbidities, living alone/with others, access to a green space, type of home, stairs to home, employment status, and COVID-19 circumstances (vaccination status, shielding, history of COVID-19 diagnosis).

#### Expected outcomes for a full trial

The below measures will be collected at baseline and again approximately 3 months and 6 months after group allocation. A data collection session will be arranged to take physical measures at Brunel University London or at participants’ homes, depending on preference and participants’ ability to travel. All questionnaire outcomes will be completed online using Qualtrics (Qualtrics, London, UK) or on paper, depending on participant preference. If it is not possible to arrange a data collection session with a participant within 3 weeks of their expected measurement date for any specific time point (e.g. due to them being uncontactable or unavailable), the data will be considered unobtainable. Similarly, this would be the case if a participant misses two scheduled data collection sessions at any given time point.

#### Sarcopenia

Objectively assessed sarcopenia is the intended primary outcome for a full trial. This will be measured in line with the European Working Group on Sarcopenia in Older People guidelines [60]. Hand grip strength will be measured using a hand grip dynamometer (Takei Scientific Instruments Co., Ltd., Niigata, Japan) on the dominant hand whilst standing with the elbow fully extended. Participants will complete three maximum attempts with a 1-min rest between each and the average recorded [61]. The Bodystat 1500 (Bodystat Ltd., Isle of Man) bioelectrical impedance device will be used to estimate muscle mass to the nearest 0.1 kg. Participants will be required to fast for 4 h prior to this measurement, avoid exercise in the 12 h beforehand and avoid caffeine and alcohol for 24 h beforehand to increase validity of the data [62]. Individuals fitted with a pacemaker will be excluded from bioelectrical impedance measures. Physical performance will be measured using the Short Physical Performance Battery (SPPB), which includes standing balance, walking speed and rising from a chair [63]. Standing balance will be tested using side-by-side, semi-tandem and tandem stands in this order for 10 s each. An 8-foot walking course will be used to evaluate walking speed with participants instructed to “walk to the other end of the course at your usual speed, just as if you were walking down the street to go to the shop”. The walk will be timed and will be performed twice with the average time used for analysis. The rising from a chair task will be completed using a straight-backed chair placed next to a wall. Participants will fold their arms and be instructed to stand up once from the chair. If this is performed successfully, participants will be asked to stand up and sit down as quickly as possibly five times, which will be timed. Each of the SPPB tests are scored on a 0–4 scale following published guidelines [63].

In addition to objective measures, the SARC-F (strength, assistance with walking, rising from a chair, climbing stairs, and falls) questionnaire will be used for participants to self-report their ability to carry a heavy load, walking, rising from a chair, climbing stairs, and falls frequency [64]. The SARC-F has been validated for sarcopenia classification that has comparable predictive ability to international diagnostic criteria [60].

#### Self-reported physical function

Activities of daily living will be assessed using the validated Groningen Activity Restriction Scale [65], which asks participants to rate their level of independence for 18 frequent daily activities, e.g. dressing yourself, going up and down stairs, and making the bed.

#### Height, weight, and body fat

Height will be measured using a portable stadiometer (Seca 213; Seca GmbH, Hamburg, Germany) to the nearest 0.1 cm with participants positioned in the Frankfurt plane. Weight will be measured in minimal clothing and no shoes using electronic weighing scales (Seca 875; Seca GmbH, Hamburg, Germany) to the nearest 0.1 kg. Body fat % and fat mass will be measured using the Bodystat 1500 to the nearest 0.1% and 0.1 kg, respectively.

#### Sitting, standing, and stepping outcomes

Following each data collection session, participants will wear an activPAL4 device on their right thigh for seven full consecutive days to provide a valid and reliable measure of sitting, standing, stepping and breaks in sitting [66, 67]. A nitrile sleeve and medical dressing (Hypafix transparent; BSN Medical Limited, Hull, UK) will be wrapped around the activPAL device to waterproof it before attaching it to their right thigh with a Hypafix dressing. A researcher will assist participants with attaching the device to their thigh and provide them with a copy of the diary during their measurement session. A guidance document and video will also be provided advising participants on how to attach the device in case they need to re-attach it during the monitoring period. Participants will be asked to wear the activPAL continuously during the monitoring period and only remove it if they go swimming to avoid it being lost. Wake and sleep times will be recorded by participants in a diary in addition to any times they undertook paid work or non-wear of the activPAL. Participants will be informed they can contact the research team to discuss any issues while completing this measure. A pre-paid envelope will be provided for participants to post the device back to the research team.

#### Mood, wellbeing, and quality of life outcomes

Sarcopenia-specific quality of life will be measured using the validated SarQoL questionnaire [68], which measures quality of life across seven domains: (1) physical and mental health, (2) locomotion, (3) body composition, (4) functionality, (5) ADL, (6) leisure activities, and (7) fears. The SarQol is scored using an automated code available from www.sarqol.org. Health service use, prescription use and pain relief medication use will be self-reported using a modified version of the Client Service Receipt Inventory [69]. The feasibility of collecting these measures is important to inform a cost-effectiveness evaluation in a full trial. The Positive and Negative Affect Schedule will be used to measure positive and negative mood [70]. Subjective wellbeing will be measured using the Office for National Statistics 4-item scale [71].

#### Process evaluation

A process evaluation, informed by previous research [72, 73], will be conducted throughout the trial to understand factors affecting how the intervention functions, mechanisms explaining its impact and implementation of the trial overall [74]. Questionnaires with scaled, closed and open questions will be completed by intervention participants to explore their experiences with each of the intervention components, barriers and strategies for sitting less, and any other behavioural changes during the study. Control and intervention participants will be asked questions regarding their experiences of completing the study measurements and whether this affected their sitting time or other behaviours. Individuals who withdraw during the study will be invited to complete a questionnaire with closed and open questions to explore the reasons for their withdrawal.

Semi-structured individual interviews will be conducted by video or phone call with a subset of intervention and control participants. Consent to take part in these interviews will be taken at the point of consent for the study. The interviews will explore reasons for taking part in the study in addition to acceptability of the study measurements and if completing these measures led to any changes in behaviour. Interviews with control participants will also explore acceptability of being randomised to this group. For intervention participants, the interviews will evaluate intervention acceptability guided by a theoretical framework for evaluating acceptability of healthcare interventions [75]. It is anticipated that interviews will last for 30–45 min each. Interviews with participants will be conducted until no new data areas are identified and saturation is deemed to have been reached, which is expected to occur with approximately 13 participants from each group [76]. All of the intervention’s health coaches (*n* = 5) will be interviewed to evaluate feasibility and fidelity of the sessions they deliver in the context of the GROW model, using motivational interviewing, assessing capability, opportunity and motivation influences on behaviour, and delivery of the intended BCTs. The number of health coaching sessions that the participants complete will also be recorded. Individuals who were eligible but did not volunteer to take part and those who dropped out during the study will be invited to complete a questionnaire to explore the reasons for this.

#### Participant incentives

Each participant will receive a £10 shopping gift voucher at each data collection time point if they take part in the data collection procedures and return the activity monitor to the research team. Travel expenses will also be reimbursed for visits made to the university for data collection.

### Data analysis

Feasibility analysis will include calculating rates for participant eligibility (number eligible/number assessed for eligibility × 100), recruitment (number randomised/number eligible × 100), retention (number who complete measurements at 3 and 6 months/number enrolled into study × 100), and missing data (number of complete datasets for outcome measures/number of participants enrolled × 100). To assess trial safety, frequency of falls, hospital admissions and adverse events will be calculated for each group in addition to exploring trends in pain and fatigue (using mean ± SD).

All interviews will be transcribed verbatim using the automated transcription feature of Microsoft Teams (Microsoft Corporation, WA, USA) or a transcribing software (Otter.ai; CA, USA) for interviews conducted by other means, e.g. phone call. Transcripts will be subsequently checked for accuracy by the research team. The Framework Method [77] will be used to analyse this data. This will include deductive and inductive coding to generate themes related to positive and negative participant experiences in relation to intervention acceptability, the intervention’s active ingredients, appropriateness of data collection procedures and factors affecting implementation of the trial. Quotes will be used to illustrate these themes. Deductive coding will be used to evaluate the intervention in the context of the theoretical framework for acceptability of healthcare interventions [75]. This framework explores acceptability in the context of affective attitude, burden, ethicality, intervention coherence, opportunity costs, perceived effectiveness, and self-efficacy. Inductive coding will allow for data-driven insights and contextual factors to be explored. Intervention fidelity will be explored in terms of participant and health coach adherence and engagement with the intervention in relation to the planned protocol. Rating scales within the process evaluation questionnaires will be descriptively analysed based on means, SD, and frequencies.

The potential efficacy of the intervention for improving the intended primary (sarcopenia) and secondary outcomes (physical function; sarcopenia-specific quality of life; daily and prolonged sitting, breaks in sitting, standing, and stepping; mood, wellbeing and quality of life) in a definitive trial will be analysed using descriptive statistics. This will include mean ± SD as well as median and interquartile range for continuous data. Frequency, counts, and percentages will be used for categorical data.

### Progression criteria to a definitive trial

Progression from this feasibility trial to a definitive full RCT will be based on the following criteria:

- The required number of participants are recruited in the intended timeframe.
- At least 70% of the participants complete the study and provide valid primary outcome data (sarcopenia) at all time points.
- The intervention is deemed to be acceptable and safe to the participants.
- The intervention is delivered as planned.

In the case that these criteria are not met, factors affecting implementation of the trial will be explored to identify potential issues that can be adapted in preparation for a full trial.

### Data management

Personal data collected in this study will be handled and stored in line with the U.K. Data Protection Act (2018). Electronic data will be stored on computers that are password protected and online using Qualtrics which is a secure password protected platform. Hard copy data will be stored securely at Brunel University London. Only members of the immediate research team will have access to the data. Participants will be assigned a participant ID number when they enrol into the study, which will be used on all data collection sheets and data files to maintain anonymity. Data collected during measurement sessions will be directly entered into Qualtrics. Interview recordings will be stored securely on Microsoft Teams or an encrypted Dictaphone and will be deleted following data analysis. Qualitative data will be managed using NVivo software (QSR International, Doncaster, Australia). All raw quantitative data will be checked for quality using range checks prior to analysis.

### Trial monitoring

A Trial Steering Committee (TSC) comprising of an independent Chair, an independent expert in the study area, public members, the principal investigator, and co-investigators will monitor progress of the project, protocol adherence, substantial protocol amendments, and participant safety. The TSC will also advise the investigators on all aspects of the project.

The TSC will meet every 3 months. A data monitoring committee is not needed due to the feasibility nature of the study.

### Dissemination strategy

The findings will be disseminated to the public via newsletters distributed to the community, a dedicated University webpage, social media, and a virtual public webinar. Dissemination to academic and practitioner audiences will be via publication in scientific journals and presentation at national and international conferences. All data and dissemination materials will be made open access to maximise reach and impact of the findings.

## Discussion

Older adults living with frailty have a significantly increased risk of losing independence, unplanned hospitalisation and nursing home admission [8]. These risks increase markedly in individuals progressing from mild to moderate and severe levels of frailty [8]. Sarcopenia is a key factor in the development and progression of frailty and is adversely associated with muscle mass, physical function, and the ability to carry out activities of daily living in community-dwelling older adults [4, 5]. Observational evidence suggests that higher volumes of sedentary time are associated with increased risk of sarcopenia and impaired physical function [11, 12]. However, there is a paucity of research evaluating the feasibility, safety, and acceptability of sedentary behaviour interventions for improving sarcopenia and independent living in older adults. This study will address this gap in knowledge, specifically in community-dwelling older adults living with frailty. The findings will improve our understanding of the feasibility, safety, and acceptability of remotely delivered interventions in this population group and inform their suitability for supporting older adults with frailty in reducing and breaking up sitting. In turn, findings could have wider implications for health promotion in a range of social distancing and isolation scenarios such as during pandemics and severe weather, economic constraints, travel and access difficulties, and social isolation. If indicated, the findings will inform a definitive RCT to determine the effectiveness and cost-effectiveness of the intervention for improving sarcopenia and independent living in older adults living with frailty, and could inform public health and clinical care guidelines.

## Supplementary Information

**Additional file 1.**

**Additional file 2.**

## Data Availability

Not applicable.

## Abbreviations

ADL: Activities of daily living
APEASE: Acceptability, Practicability, Effectiveness/cost-effectiveness, Affordability, Safety/side-effects, Equity
BCT: Behaviour change technique
BCW: Behaviour Change Wheel
COM-B: Capability, Opportunity, Motivation-Behaviour
GROW: Goals, Reality, Options, Will
Frail-LESS: LEss Sitting and Sarcopenia in Frail older adults
OARS: Open-ended questions, Affirmations, Reflective listening, Summaries
PACE: Partnership, acceptance, compassion and evocation
RCT: Randomised controlled trial
RULE: Resist the righting reflex; Understand client motivation; Listen; Empower
SARC-F: Strength, assistance with walking, rising from a chair, climbing stairs, and falls
SPPB: Short Physical Performance Battery
TSC: Trial Steering Committee
