# Supplementary material for: The Frail-LESS (LEss Sitting and Sarcopenia in Frail older adults) intervention to improve sarcopenia and maintain independent living via reductions in prolonged sitting: a randomised controlled feasibility trial protocol
Source: Pilot Feasibility Stud. 2023 Jan 7;9:1. doi: 10.1186/s40814-022-01225-7 (PMC9823257; doi:10.1186/s40814-022-01225-7)
Supplement: Supplementary file 1 — Additional file 1. [file 40814_2022_1225_MOESM1_ESM.docx]

**
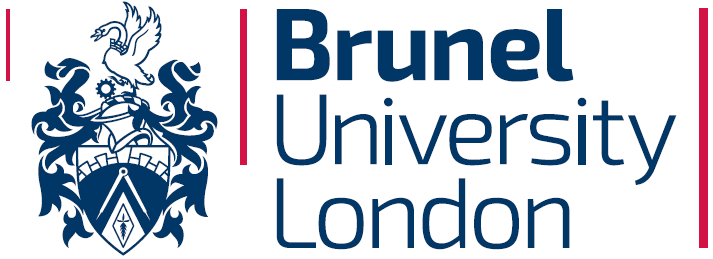
CONSENT FORM**


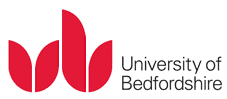


College of Health, Medicine and Life Sciences

Department of Life Sciences

**The Frail-LESS study**

APPROVAL HAS BEEN GRANTED FOR THIS STUDY TO BE CARRIED OUT FROM 01^st^ SEPTEMBER 2021

| **The participant should complete the whole of this sheet.** | | |
| --- | --- | --- |
| **PLEASE INITIAL EACH BOX** | | |
| I have read and understood the Participant Information Sheet | |  |
| I have had an opportunity to ask questions and discuss this study | |  |
| I have received satisfactory answers to all my questions | |  |
| I understand that I will not be referred to by name in any report concerning this study | |  |
| I understand that: | | |
| - I am free to withdraw from this study at any time - I don’t have to give any reason for withdrawing - Choosing not to participate or withdrawing will not affect my rights or my care | |  |
| I agree to my interview being audio recorded if I participate in the interview part of the study | |  |
| I agree to the use of non-attributable quotes when the study is written up or published | |  |
| The procedures regarding confidentiality have been explained to me | |  |
| I agree that my anonymised data can be stored and shared with other researchers for use in future projects. | | Y N |
| I agree to my data being shared with my health coach If I am entered into the intervention group so that they are able to contact me | |  |
| I agree to being asked to complete a withdrawal questionnaire if I decide to stop taking part in the study | | Y N |
| I agree to take part in this study | |  |
| I agree to my GP being notified of my participation in this study | |  |
| GP name:___________________________________________________________  GP address:_________________________________________________________  __________________________________________________________________ | | |
| **PLEASE TURN OVER** | | |
| Address of research participant: | | |
| Email address: | | |
| Phone number: | | |
| Signature of research participant: | | |
| Print name: | Date: | |
|  |  | |
| Consent received by (researcher signature): | | |
| Researcher’s name: | Date: | |

Consent form storage: 1 copy to the participant, 1 copy for research file
